# Phenotypic characterization of frontal cortex microglia in a rat model of post‐traumatic stress disorder

**DOI:** 10.1002/brb3.2011

**Published:** 2021-01-12

**Authors:** Thomas S. Cotrone, Charina B. Hocog, Joseph T. Ramsey, Marcus A. Sanchez, Heather M. Sullivan, Angus G. Scrimgeour

**Affiliations:** ^1^ Military Nutrition Division U.S. Army Research Institute of Environmental Medicine Natick MA USA; ^2^ Veterinary Support and Oversight Branch U.S. Army Research Institute of Environmental Medicine Natick MA USA

**Keywords:** Fear Extinction, Microglia, Post‐traumatic Stress Disorder, PTSD, Rat, Single Prolonged Stress, SPS

## Abstract

**Introduction:**

Post‐traumatic stress disorder (PTSD) is an anxiety disorder induced by psychologically traumatic events. Using a rat model, this study aimed to determine whether psychological trauma alters relative expression between pro‐inflammatory and anti‐inflammatory markers in microglia. To meet this goal, expression of genes encoding i‐NOS, arginase, TNF‐α, interleukin‐10, CD74, and Mannose Receptor C was analyzed on multiple days following trauma exposure.

**Methods:**

Single‐prolonged stress (SPS) was used to model PTSD in male Sprague‐Dawley rats. Twenty‐four rats (12 Controls and 12 SPS‐exposed) were sacrificed on Days 1, 3, and 7 post‐SPS. Twenty‐four (12 Controls and 12 SPS‐exposed) additional rats were exposed to classical fear conditioning on Day 7, and fear extinction on Days 8, 9, 10, 15, 16, and 17. Freezing behavior was measured to assess fear resolution. Microglial isolates were collected from the frontal cortex, and RNA was extracted. Changes in relative expression of target genes were quantified via RT‐PCR.

**Results:**

SPS rats showed significant decreases in IL‐10 and TNF‐α expression and increases in the i‐NOS:Arginase and TNF‐α:IL‐10 ratios compared to Controls on Day 1, but not on Day 3 or Day 7 for any of the dependent variables. Day 17 SPS rats showed a significant decrease in IL‐10 expression and an increase in the TNF‐α:IL‐10 ratio, further characterized by a significant inverse relationship between IL‐10 expression and fear persistence.

**Conclusion:**

Psychological trauma impacts the immunological phenotype of microglia of the frontal cortex. Consequently, future studies should further evaluate the mechanistic role of microglia in PTSD pathology.

## Graphical abstract

This study tested the hypothesis that exposure to psychological trauma would result in increased expression of pro‐inflammatory markers relative to anti‐inflammatory markers by microglia of the frontal cortex. To do this, frontal cortices of rats were collected at various time points after exposure to a psychological trauma in the form of Single‐Prolonged Stress. A significant change in microglial gene expression was found that correlated with key behavioral changes, indicating a possible immunological basis for post‐traumatic stress disorder.

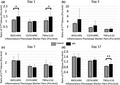

## 1 INTRODUCTION

Stress is a complex physiological and psychological response to aversive conditions (Suri & Vaidya, 2015; Yehuda & McEwen, 2004). The long‐term effects of the stress response on an individual can vary from adaptive to maladaptive depending on the characteristics of the stressful event experienced (Suri & Vaidya, 2015; Yehuda & McEwen, 2004). Post‐traumatic stress disorder (PTSD) is an example of a maladaptive anxiety disorder that can develop in individuals exposed to a psychologically traumatic stressor (American Psychiatric Association, 2013). PTSD is characterized by four symptom clusters: (a) persistent, intrusive re‐experience of the trauma, (b) avoidance of trauma‐related stimuli, (c) negative alterations in cognition and mood, and (d) alterations in arousal and reactivity (American Psychiatric Association, 2013). Diagnosis is dependent on these symptoms persisting for more than 1 month, being significant enough to create distress or functional impairment, and not being due to extraneous factors such as other mental illness or drug abuse (American Psychiatric Association, 2013). The American Psychiatric Association currently offers strong recommendations that the treatment for PTSD should be psychotherapies like cognitive behavioral therapy (CBT) (American Psychiatric Association, 2017). These therapies have been considered to have the best balance of efficacy, benefit‐to‐harm balance, patient value/preference, and applicability (American Psychiatric Association, 2017). Unfortunately, even with these strengths, the recovery rate of patients receiving these treatments can vary dramatically. For example, CBT has recovery rates reported to vary between ~40% and ~80% (Morkved et al., 2014). In order to improve recovery rates, new therapies need to be developed. Unfortunately, much of the basic pathogenesis of PTSD is still unknown. This lack of understanding hampers the development of effective treatments and necessitates the study of the pathological basis of PTSD that could lead to the discovery of new therapeutic targets.

One area of research that may reveal such targets is the relationship between PTSD and immune system dysfunction. Studies have reported that patients with PTSD show signs of immune system dysregulation. For example, they exhibit reduced natural killer cell and T‐Cell activity/responsiveness as well as higher levels of circulating pro‐inflammatory cytokines including interleukin‐1β (IL‐1β), tumor necrosis factor‐α (TNF‐α), and interleukin‐6 (IL‐6) (Gill et al., 2009; Pace & Heim, 2011). Further investigation into these phenomena have revealed that epigenetic changes in genes related to immune system regulation (e.g., CXCL2, CXCL3, CCL4, and CCL5) can occur in human subjects following psychological trauma and are consistent with the presence of a pro‐inflammatory state in PTSD (Bam, Yang, Zhou, et al., 2016; Bam, Yang, Zumbrun, et al., 2016). These findings could prove relevant to PTSD pathogenesis since pro‐inflammatory cytokines like TNF‐α (Liu et al., 2017a; Pickering et al., 2005) have been shown to be detrimental to long‐term memory formation. Since studies have implicated impaired long‐term fear extinction memory as the basis for the persistent re‐experiencing of trauma associated with PTSD (Milad et al., 2008), immune system dysfunction could play an important mechanistic role in the pathogenesis of PTSD.

As the resident immune cells of the CNS, microglia are an important population of cells to analyze when characterizing the relationship between immune system dysfunction and the pathogenesis of PTSD‐associated memory impairment. Microglia play a mechanistic role in the pathogenesis of many neurological diseases/disorders including Alzheimer's disease, neuropathic pain, amyotrophic lateral sclerosis (ALS), traumatic brain and spinal cord injury, and HIV‐associated neurocognitive disorder (Burke et al., 2014; Kumar et al., 2016; Liu et al., 2017b; Martin et al., 2017; Singh et al., 2015; Tang & Le, 2016) that are associated with memory deficits. Relevant to PTSD, rodent models have demonstrated that psychological stress can promote a pro‐inflammatory state in both microglia and macrophages. For example, exposure to either inescapable shock or repeated social defeat has been shown to induce increased expression of markers of nonspecific microglial and macrophage activation (Frank et al., 2007; Wohleb et al., 2012, 2013). These stressors also primed microglia and macrophages in such a way that subsequent inflammatory challenges induced an exaggerated pro‐inflammatory response from these cell populations (Frank et al., 2007, 2012; Wohleb et al., 2012, 2013).

While the association between PTSD and immune system dysfunction is becoming increasingly more evident given recent research (Wang et al., 2017), it remains to be determined as to whether or not this change in inflammatory state plays a significant role in the predisposition, development, and/or maintenance of PTSD. As such, this study aimed to characterize the microglial inflammatory response in a rat model of PTSD in order to shed more light on the mechanistic role that microglia play in the pathogenesis of PTSD. We hypothesized that exposure to psychological trauma would result in an increase in expression of pro‐inflammatory markers relative to anti‐inflammatory markers in the microglia of the frontal cortex. To characterize this relative change, we selected six target genes associated with either a pro‐inflammatory or anti‐inflammatory microglial phenotype. These targets included the enzymes i‐NOS (pro‐inflammatory) and arginase (ARG) (anti‐inflammatory), the cytokines TNF‐α (pro‐inflammatory) and interleukin‐10 (IL‐10) (anti‐inflammatory), and the cell surface proteins CD74 (pro‐inflammatory) and Mannose Receptor C (MRC) (anti‐inflammatory). Additionally, to better understand the pathogenesis of this disorder, we aimed to characterize the time course of these changes and to determine whether they are correlated with behaviors associated with PTSD.

## 2 MATERIAL AND METHODS

### 2.1 Animals

Ninety‐six male Sprague‐Dawley rats (8–10 weeks of age, ~250 g, Charles River Laboratory, Wilmington MA) were used in this study. Male Sprague‐Dawley rats were selected for this study because they have been extensively used in the Single‐Prolonged Stress model of PTSD, and exhibit consistent behavioral and biochemical changes similar to those exhibited by humans with PTSD. Upon arrival, all rats were pair‐housed and allowed to acclimate before the start of the study. All rats had ad libitum access to water and standard rat chow (Harlan‐Teklad AIN‐79) throughout the study and were maintained on a 12:12 hr light:dark schedule (lights were on from 7 a.m. to 7 p.m.). Temperature and relative humidity of housing rooms were maintained within the ranges of 20–26°C and 30%–70%, respectively. All experimental procedures were approved by the Institutional Animal Care and Use Committee of the US Army Research Institute of Environmental Medicine and complied with standards set by the Association for Assessment and Accreditation of Lab Animal Care.

### 2.2 Single‐prolonged stress

This study utilized the Single‐Prolonged Stress (SPS) procedure as a model for PTSD (Liberzon et al., 1997). Forty‐eight rats were randomly assigned to receive SPS. SPS consisted of three consecutive stressors: restraint, forced swim, and ether anesthetization on the same day. For restraint, rats were individually placed in a plastic tail vein restraint tube (diameter = 7 cm; Braintree Scientific Inc., Braintree MA) without habituation. Rats were then restrained to the point of immobilization and remained in the device for 2 hr. Immediately following the 2 hr restraint, 4 rats at a time were placed together into a cylindrical plastic container (diameter = 30 cm; height = 60 cm; Med Associates Inc., St. Albans VT) filled with clean water (~25°C). Individual rats remained in the water for 20 min or until they could no longer keep from drowning (whichever came first). After the forced swim test, rats were dried with a towel and given 10 min to rest. After the rest period, each group of four rats from the swim test was placed in a bell jar (i.e., all four rats together) and exposed to ether fumes. Rats remained in the jar until they lost the righting reflex, and there were no responses to a tail pinch. Following anesthesia, rats were transferred to new cages and were singly housed for the rest of the experiment. The 48 rats assigned as Controls were left in their home cages throughout this process and continued to be pair‐housed. Groups of rats (12 SPS and 12 Controls per group) were sacrificed at one of four time points after exposure to SPS: Day 1, Day 3, Day 7, and Day 17 (see Figure S1).

### 2.3 Classical fear conditioning and extinction

Seven days after SPS exposure, all rats in the Day 17 group underwent classical fear conditioning. Rats were placed in sound‐attenuating chambers with clear polycarbonate walls and a stainless steel grid floor (Med Associates Inc.). The dimensions of a single chamber were 25(H) × 25 (W) × 30 (L) cm). Each chamber was illuminated with an infrared house light and cleaned with isopropanol wipes before a rat was placed in the chamber. Rats were placed into the chamber alone and allowed to explore/acclimate for 180 s. After this period, the rats were presented with an auditory tone (2 kHz, 80 dB) for 10 s. The tone coterminated with a single unconditioned fear stimulus (US) in the form of a 1‐s scrambled shock (1.0 mA) delivered through the grid floor followed immediately by a 50 s rest interval. This was repeated four additional times to yield five total tone‐shock pairings (each separated by a 50 s rest interval).

Twenty‐four hours after fear conditioning, rats from the Day 17 group began two series of fear extinction training. Each series occurred over three days and consisted of three daily extinction trials: Days 8, 9, and 10 for Series 1, and Days 15, 16, and 17 for Series 2. During each extinction trial, the rats were placed back in the conditioning chambers described above, except 70% ethanol was used to clean the chambers and plastic inserts were placed along the chamber walls in order to provide a different olfactory and visual context. After a 180‐s acclimation period, rats were presented with 8 extinction queues. Each queue consisted of presenting a conditioned stimulus (CS) in the form of a 10‐s auditory tone (2 kHz, 80 dB) with no concurrent shock delivery followed by a 50‐s rest interval. Behavior was recorded with a digital camera during the entire period. VideoFreeze software (Med Associates Inc.) was used to determine the time rats spent exhibiting freezing behavior during and after each queue. Behavioral data points were then calculated by dividing the time spent exhibiting freezing behavior by 60 s to yield “%Freezing Time” for each queue.

### 2.4 Tissue collection

Rats were placed under deep anesthesia with 5% isoflurane, and the thoracic cavity was exposed by removing the ventral rib cage. Rats were exsanguinated by severing the vena cava and infusing isotonic saline into the left ventricle of the heart until the liver and extremities blanched. Rats were then decapitated, and the brains were rapidly removed. Using a brain mold (Zivic Instruments), the brains were divided in two by making a coronal section at the level of the optic chiasm. From the caudal sample, the left and right hippocampi were dissected out, flash frozen in liquid nitrogen, and stored at −80°C. From the rostral sample, the olfactory bulbs and subcortical tissue were removed, and the remaining frontal cortex was placed on ice and used immediately for microglial isolation.

### 2.5 Microglial isolation

Frontal cortex tissue was enzymatically and mechanically homogenized using an Adult Brain Dissociation Kit (Miltenyi Biotec, 130‐107‐677; Somerville MA) and gentleMACS C‐Tubes (Miltenyi Biotec, 130‐093‐237) following the manufacturer's instructions. The resulting homogenate was run through a 70‐µm filter (Miltenyi Biotec, 130‐101‐812) to generate a single‐cell suspension. The suspension was treated with anti‐myelin antibody‐coated beads (Miltenyi Biotec, 130‐096‐733) and passed through a magnetized selection LS‐column (Miltenyi Biotec, 130‐042‐401) to remove myelin debris. The flow‐through sample was treated with anti‐CD11b/c + antibody‐coated beads (Miltenyi Biotec, 130‐105‐634) and then passed through a magnetied selection MS‐column (Miltenyi Biotec, 130‐042‐201). The isolated CD11b/c + cells bound to the MS‐columns were collected by removing the columns from the magnetic field and rinsing them with calcium‐ and magnesium‐deficient PBS (ThermoFisher Scientific). The flow‐through containing the CD11b/c + cells was stored at −80°C for later RNA isolation.

### 2.6 RNA isolation, reverse transcription, and real‐time PCR assay

The flow‐through samples from the microglial isolation process were thawed at 4°C. Total RNA was extracted from the samples using an RNeasy Kit (Qiagen, 74034; Gaithersburg MD) following the manufacturer's instructions. Total RNA concentration in each sample was determined using a NanoDrop Spectrophotometer (ThermoFisher Scientific). Reverse transcription of 0.5 µg of RNA was then performed for each sample using a SuperScript™ Reverse Transcriptase Kit (ThermoFisher Scientific, 18090010) following the manufacturer's instructions. Real‐time PCR was then performed on a StepOnePlus Real‐time PCR System (ThermoFisher Scientific) using TaqMan Fast Advanced Master Mix (Applied Biosystems, 4444964) and TaqMan Gene Assays (Applied Biosystems; 18 s, 03928990_g1; TNF‐α, Rn99999017_m1; ARG, Rn00691090_m1; MRC, 01487342_m1; IL‐10, Rn01483988_g1; NOS2, Rn00561646_m1; CD74, Rn00565062_m1). Duplicates of each sample were run under the following parameters: 50°C for 2 min (incubation), 95°C for 10 min (activation), 95°C for 15 s (denaturation) followed by 60°C for 1 min (annealing and extension) (40 cycles of denaturation/annealing/extension). Gene expression was calculated relative to the endogenous Control (18 s) to determine relative expression values (2^−ΔCt^, where the Ct value is the threshold cycle). To evaluate absolute changes in gene expression, means of the 2^−ΔCt^ values were used as dependent variables for ARG, i‐NOS, IL‐10, TNF‐α, MRC, and CD74. To evaluate relative changes in pro‐inflammatory/anti‐inflammatory state, ratios of the 2^−ΔCt^ values were used as dependent variables to report i‐NOS:ARG, TNF‐α:IL‐10, and CD74:MRC ratios.

#### 2.6.1 ELISA

Whole hippocampi from Day 17 rats were thawed and minced in 1 ml of ice cold PBS. The PBS/tissue mixtures was then homogenized in gentleMACS C‐Tubes (Miltenyi Biotec, 130‐093‐237). Homogenized samples were next exposed to two freeze‐thaw cycles at −80°C/4°C. Samples were then centrifuged for 15 min at 1500 *g*, and the supernatant was collected. Following the manufacturer's instructions, a Rat Glucocorticoid Receptor ELISA Kit (MyBioSource, MBS752565; San Diego CA) was used to quantify glucocorticoid receptor (GR) expression in the supernatant. Protein concentration was measured by spectrophotometry on a SpectraMax M3 (Molecular Devices; Sunnyvale CA).

### 2.7 Statistical analysis

All statistical analyses were performed using SPSS Statistics software, Version 26 (IBM), and results were expressed as group means ± standard deviations. Tests for normality (Shapiro—Wilk's) were performed on each treatment group (SPS/Control) within each time point (Day 1/Day 3/Day 7/Day 17) to ensure that all data sets were normally distributed and that subsequent parametric analyses could be used. To determine differences in fear expression during CFC, two‐way repeated measures ANOVA was performed with unconditioned stimulus (US) as a within‐subjects factor and SPS‐exposure status as a between‐subjects factor. To determine differences in fear expression during Fear Extinction, two‐way repeated measure ANOVA was performed with “Day” as a within‐subjects factor and SPS‐exposure status as a between‐subjects factor. Differences between the means of the changes in gene expression (2^−ΔCt^ values and 2^–ΔCt^ ratios), GR protein expression, and %Freezing Time were evaluated using one‐way ANOVA (significance threshold was *p* < .05). Correlation between gene expression and fear behavior was evaluated using the Pearson Correlation.

## 3 RESULTS

### 3.1 SPS‐induced impairment of fear extinction memory

**Figure 1:**
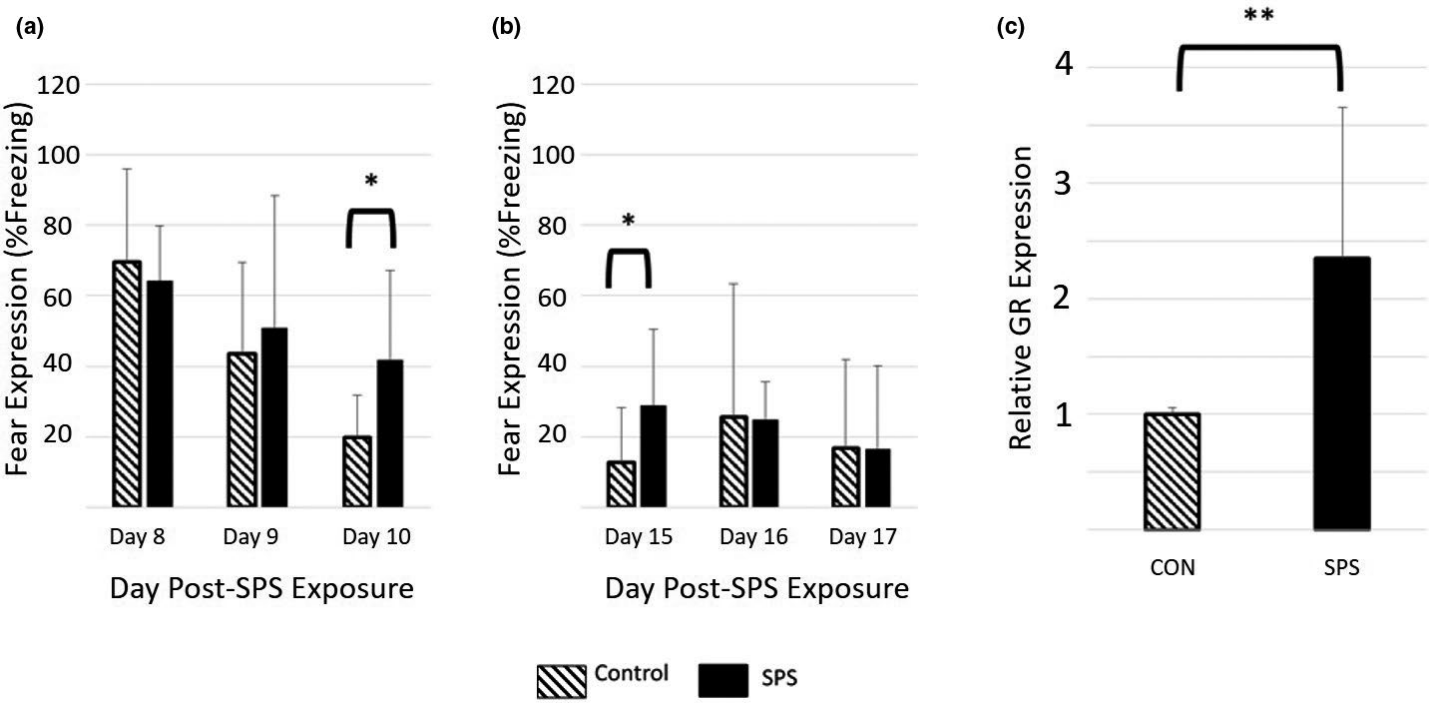
Validation of the SPS Model by Evaluating Persistence of Fear Behavior and Expression of Hippocampal GR. (a) First extinction series for Day 17 rats. SPS induced a significant increase in expression of fear behavior by the end of the first extinction series (Day 10). (b) Second extinction series for Day 17 rats. SPS induced a significant increase in expression of fear behavior at the beginning of the second extinction series (Day 15). (c) Hippocampal GR expression for Day 17 rats. GR expression was significantly increased in SPS rats on Day 17 compared to Controls. Taken together, these data suggest that the SPS rats in this experiment are exhibiting behavioral and biochemical changes that are characteristic of a PTSD model. Data = mean ± *SD*; *n* = 12 rats per group; *p*‐values < .05 (*), <.01 (**)

Similar to human patients with PTSD, rats exposed to SPS exhibit impaired fear extinction memory (Milad et al., 2006; Norrholm & Jovanovic, 2018). In this study, we used this behavioral outcome as one of two ways to validate our model. More specifically, rats in the Day 17 group were exposed to a classical fear conditioning paradigm on Day 7, and fear expression during two subsequent fear extinction series (Series 1: Days 8–10; Series 2: Days 15–17) was evaluated. These behavioral data were then analyzed to assess the retention of fear extinction memories over time. Two‐way repeated measures ANOVA revealed a significant interaction between Day and SPS‐exposure (*p* < .01, *F* = 12.318, *df* = 23, partial Eta^2^ = 0.560). Post hoc analysis revealed significantly higher expression of freezing behavior in SPS rats on Day 10 (Figure 1a, Table S1; *p* < .05, *F* = 7.805, *df* = 23, partial Eta^2^ = 0.262) and Day 15 (Figure 1b, Table S1; *p* < .05, *F* = 4.333, *df* = 23, partial Eta^2^ = 0.165). To rule out the possibility that these group behavioral differences were due to SPS‐induced effects on establishment of fear memory during CFC, a two‐way repeated measures ANOVA was performed with unconditioned stimulus (US) number as a within‐subjects factor and SPS‐exposure status as a between‐subjects factor. This analysis failed to show a significant effect of SPS on fear expression during CFC (Figure S2). To also rule out the possibility that these behavioral changes were the result of impaired fear extinction memory formation within individual extinction trials, average fear expression of the first four CS presentations (i.e., Early Extinction) was compared to that of the last four CS presentations (i.e., Late Extinction) on each of Days 8, 9, 10, 15, 16, and 17. A two‐way repeated measures ANOVA was then performed with “time within trial” (i.e., Early Extinction or Late Extinction) as a within‐subjects factor and SPS‐exposure status as a between‐subjects factor. This analysis showed no significant interaction between SPS‐exposure and time within a trial, indicating that SPS did not alter the rate of extinction within any given extinction trial (Figures S3 and S4). Taken together, these findings are consistent with other studies using the SPS model (Eagle et al., 2013; Knox et al., 2012), and indicate that the SPS rats in this study exhibited impaired fear extinction memory compared to Controls.

### 3.2 Effect of SPS on hippocampal GR expression

Multiple studies have shown that SPS increases hippocampal GR expression in rats (Eagle et al., (2013); Knox et al., 2012; George et al., 2013). As such, this biomarker was used as a second means of validating the SPS model in this study. Following sacrifice on Day 17, whole hippocampi were collected from each rat, and ELISA was used to measure GR expression. One‐way ANOVA revealed a significant increase in GR expression in SPS rats compared to Controls (Figure 1c, Table S2; *p* < .01, *F* = 12.600, *df* = 23, partial Eta^2^ = 0.364). This finding indicates that the rats exposed to SPS in this study exhibited biochemical changes that are characteristic of the SPS model.

### 3.3 Effect of SPS on frontal cortex microglia biomarker expression

The objective of this study was to determine whether the inflammatory phenotype of microglia can be altered by SPS exposure. To that end, the expression for each of three pro‐inflammatory markers and three anti‐inflammatory markers was measured in microglial isolates from the frontal cortex. It is generally accepted that many of the SPS‐induced behavioral changes do not develop until seven days after exposure (Lisieski et al., 2018). As such, microglial samples were collected at three time points (i.e., Day 1, Day 3, and Day 7) during this seven‐day period in order to evaluate microglial phenotypes as symptoms developed. A fourth time point (Day 17) was included in the experimental design as a means of evaluating the changes in microglial phenotype that exist while the behavioral and biochemical changes were expressed.

**Figure 2:**
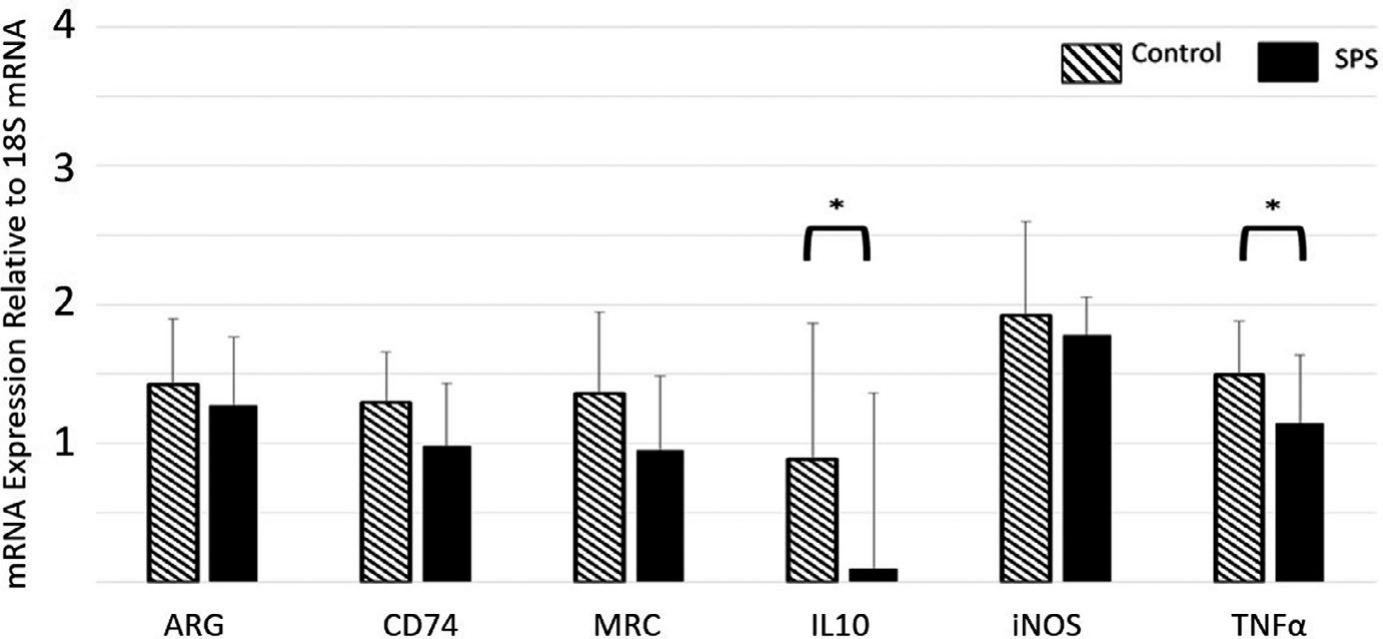
Relative Gene Expression in Microglia of the Frontal Cortex (Day 1). On Day 1, microglia from SPS‐exposed rats exhibited significantly lower expression of both IL‐10 and TNF‐α compared to those from Controls. Data = mean ± *SD*; *n* = 12 rats per group; *p*‐values < .05 (*)

**Figure 3:**
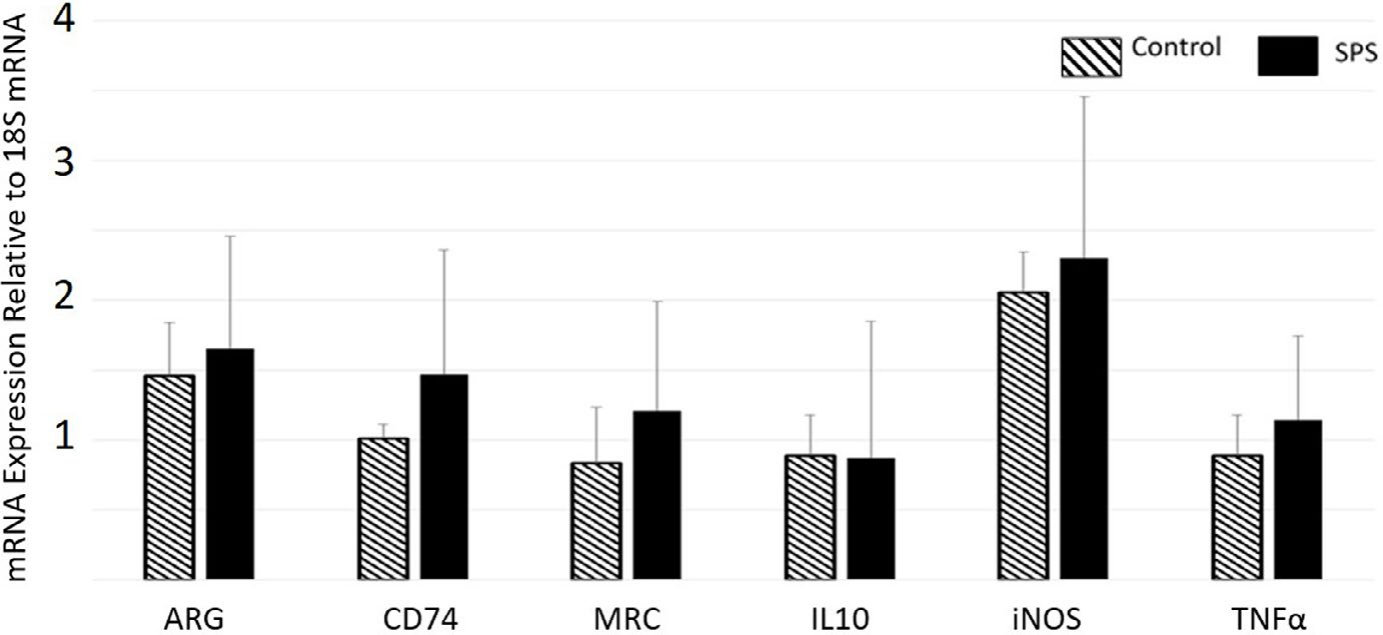
Relative Gene Expression in Microglia of the Frontal Cortex (Day 3). On Day 3, microglia from SPS‐exposed rats did not exhibit significant differences in expression of any of the target genes compared to those from Controls. Data = mean ± *SD*; *n* = 12 rats per group

**Figure 4:**
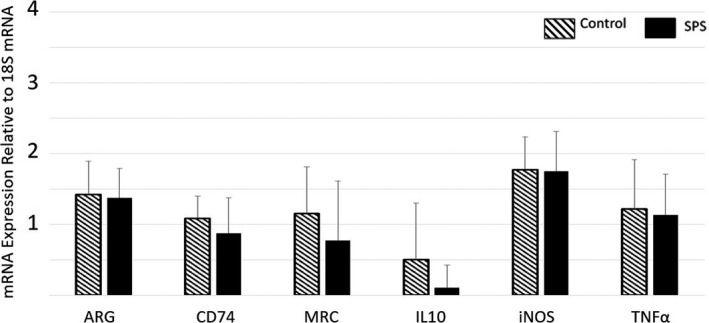
Relative Gene Expression in Microglia of the Frontal Cortex (Day 7). On Day 7, microglia from SPS‐exposed rats did not exhibit significant differences in expression of any of the target genes compared to those from Controls. Data = mean ± *SD*; *n* = 12 rats per group

**Figure 5:**
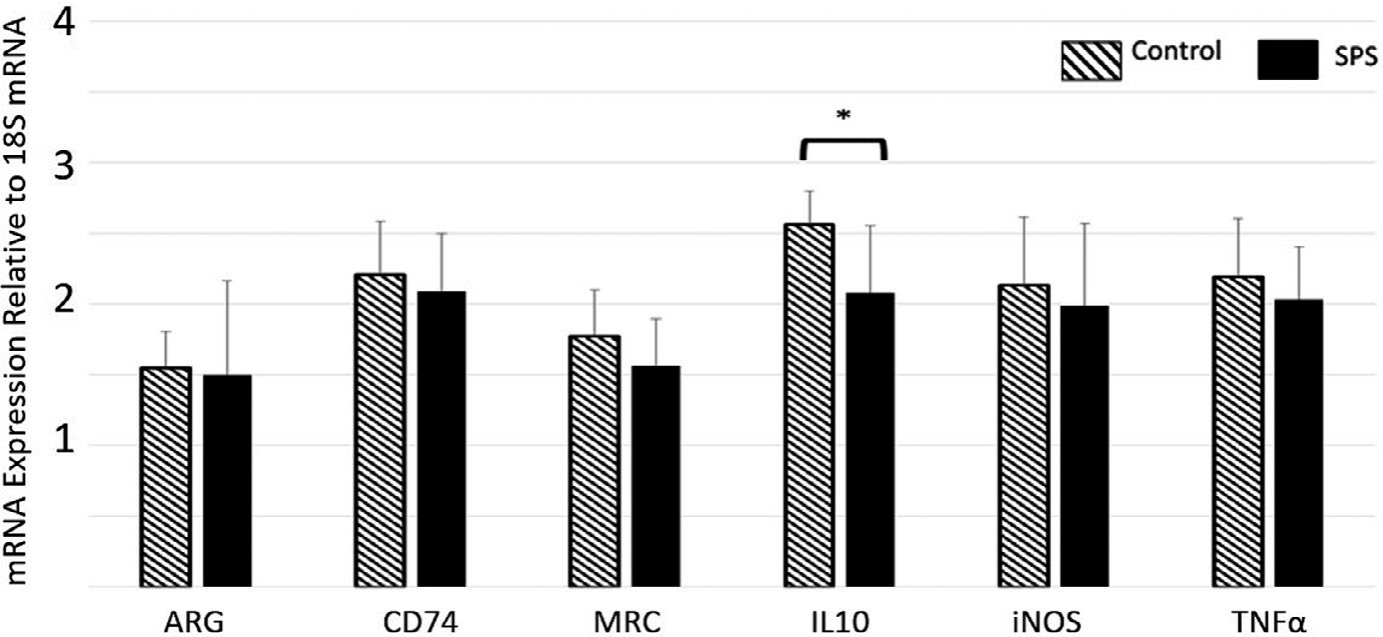
Relative Gene Expression in Microglia of the Frontal Cortex (Day 17). On Day 17, microglia from SPS‐exposed rats exhibited significantly lower IL‐10 expression compared to those from Controls. Data = mean ± *SD*; *n* = 12 per group; *p*‐values < .01 (*)

For rats in the Day 1 group, SPS induced a significant decrease in expression of TNF‐α (*p* < .05, *F* = 4.406, *df* = 23, partial Eta^2^ = 0.167) and IL‐10 (*p* < .05, *F* = 3.721, *df* = 23, partial Eta^2^ = 0.1645) compared to Controls (Figure 2, Table S2). There were no significant differences in biomarker expression between the SPS and Control rats on Day 3 or Day 7 (Figure 3 and 4, respectively, Table S2). For rats in the Day 17 group, SPS induced a significant decrease in expression of IL‐10 alone (*p* < .01, *F* = 9.977, *df* = 23, partial Eta^2^ = 0.296) (Figure 5, Table S2).

### 3.4 Effect of SPS on ratios of microglia biomarker expression

**Figure 6:**
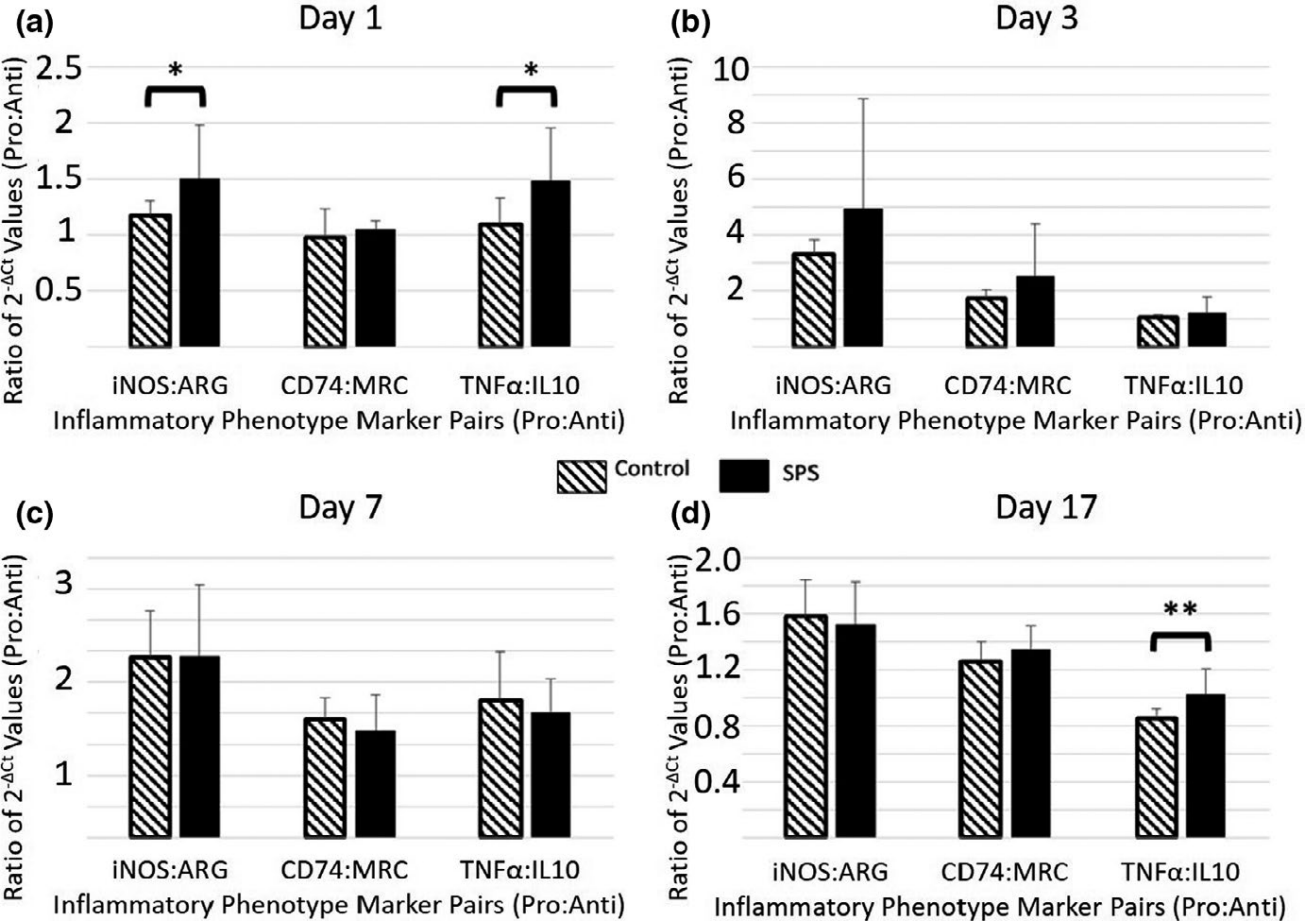
Comparison of Biomarker Ratios (Days 1, 3, 7, and 17). (a) Comparison of biomarker ratios (i‐NOS:ARG, CD74:MRC, TNF‐α:IL‐10) for Day 1 rats. SPS induced a significant increase in both the i‐NOS:ARG and TNF‐α:IL‐10 ratios. (b) and (c) Comparison of biomarker ratios for Day 3 and Day 7 rats, respectively. No significant differences in biomarker ratios were demonstrated on Days 3 and 7. (d) Comparison of biomarker ratios for Day 17. SPS induced a significant increase in the TNF‐α:IL‐10 ratio alone. Data = mean ± *SD*; *n* = 12 rats per group; *p*‐values < .05 (*) and < 0.01 (**)

Studies have shown that there is a wide variety in microglial responses to various stimuli (Ransohoff, 2016). Moreover, there can be considerable overlap of upregulated and/or downregulated gene and protein expressions between these responses (Ransohoff, 2016). Due to this complexity, simply measuring the change in expression of individual genes may not allow for meaningful descriptions of microglial activity. To accommodate for this, we evaluated how the pro‐inflammatory and anti‐inflammatory target genes changed in relation to each other following SPS exposure. To do this, we calculated ratios of TNF‐α:IL‐10, CD74:MRC, and i‐NOS:ARG to represent the Pro‐inflammatory:Anti‐inflammatory (Pro:Anti) relationships for cytokines, surface proteins, and cytoplasmic enzymes, respectively. The results of this study showed that SPS induced an increase in the i‐NOS:ARG (*p* < .05, *F* = 5.631, *df* = 23, partial Eta^2^ = 0.201) and TNF‐α:IL‐10 (*p* < .05, *F* = 7.311, *df* = 23, partial Eta^2^ = 0.249) ratios in Day 1 rats (Figure 6a). However, no significant differences were demonstrated on Days 3 or 7 (Figure 6b,c, respectively). A significant increase in the TNF‐α:IL‐10 ratio alone (*p* < .01, *F* = 9.210, *df* = 23, partial Eta^2^ = 0.295) was demonstrated in Day 17 rats (Figure 6d).

### 3.5 Relationship between microglial phenotype and impaired fear extinction memory expression

**Figure 7:**
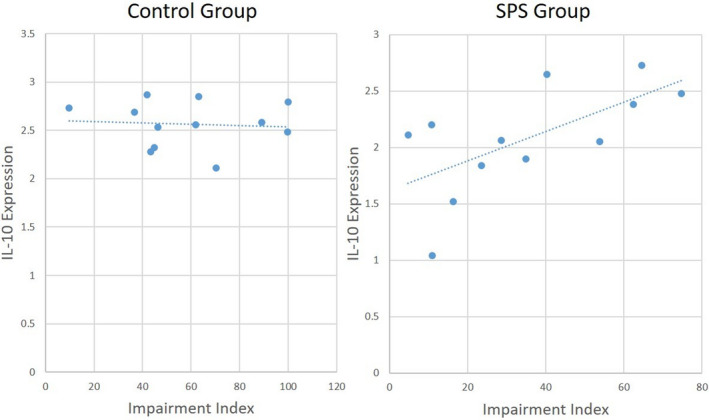
Correlation between IL‐10 Expression and Impaired Fear Extinction Memory Retention. To evaluate the potential functional connection between decreased IL‐10 expression and impaired fear extinction memory in SPS rats, a Pearson correlation was performed. An “impairment” index was calculated for each rat by subtracting %Freezing during the first trial of the second extinction series (i.e., Day 15) from the first trial of the first extinction series (i.e., Day 8). For interpretation purposes, it should be noted that when fear expression on Days 8 and 15 have similar values (an indication of poor fear extinction memory retention), the index values will become smaller. Thus, small index values correspond with reduced fear extinction memory retention. A significant positive correlation (*p* < .05, *R* = 0.645) was found in the SPS group (Right) between IL‐10 expression and the Impairment index, suggesting that low IL‐10 levels are correlated with low index values (i.e., low IL‐10 expression is correlated with impaired fear extinction memory retention). No significant correlation was found in the control group (Left).

A secondary goal of this study was to evaluate the relationship between microglial phenotype and impaired fear extinction memory expression following SPS exposure. To accomplish this, post hoc Pearson Correlation was used to compare an index of impaired fear extinction memory (i.e., “impairment” index) to IL‐10 expression. This index was calculated for each rat by subtracting %Freezing during the first trial of the second extinction series (i.e., Day 15) from the first trial of the first extinction series (i.e., Day 8). Among control rats (Figure 7a), there was no significant correlation between IL‐10 and the “impairment” index. However, among SPS‐exposed rats (Figure 7b), a statistically significant (*p* < .05) positive (0.645) correlation between IL‐10 expression and the “impairment” index was found.

## 4 DISCUSSION

### 4.1 Validation of the SPS model

The first step of this experiment was to validate that we successfully produced a PTSD model using SPS exposure. To accomplish this goal, we evaluated a behavioral outcome and a biochemical outcome commonly reported as characteristic indicators of the SPS model. Regarding behavior, studies have demonstrated that SPS causes a state of impaired fear extinction memory expression due to a failure of fear extinction memory retention (Knox et al., 2012, 2016). For biochemical changes, increased expression of hippocampal GR is a commonly reported molecular change in SPS‐exposed rats (Eagle et al., 2013; Knox et al., 2012; Liberzon et al., 1999). While the exact relationship between increased GR expression and the SPS phenotype has yet to be fully elucidated, there is evidence to support the use of GR expression for validation of the SPS model. For example, Knox et al. (2012) demonstrated that exposure to partial SPS (i.e., SPS with one of the model's three stressors excluded) induced an increase in hippocampal GR expression. However, the increase induced by partial SPS was significantly less than that induced by full SPS, suggesting a positive association between the level of stress experienced in the model and hippocampal GR expressed in the hippocampus (Knox et al., 2012). Furthermore, Eagle et al. (2013) suggested a potential mechanistic link between GR expression and the SPS phenotype by showing that the increased GR in the hippocampus expression following SPS exposure is accompanied by an increase in phosphorylated Akt (a protein kinase linked to stress‐induced anxiety behaviors). As such, we selected these two factors for the purpose of model validation. Our results revealed that SPS‐exposed rats exhibited significantly elevated %Freezing times during both extinction series. Our analysis of fear behavior failed to show any significant effect of SPS on the formation of fear memory with CFC. Likewise, we failed to show any significant effect of SPS on fear extinction within each extinction trial. Taken together, these findings indicate SPS‐induced impairment of fear extinction memory retention over some 24‐hr periods. Hippocampal GR expression was also significantly elevated in the SPS group compared to that of the Control group. Together, these findings are consistent with previous studies that have used SPS, and thus indicate that the rats in this study were showing behavioral and biochemical signs that would be expected for a rodent model of PTSD. As such, we concluded that SPS in this study successfully induced a valid PTSD‐like state in the SPS‐exposed rats.

### 4.2 SPS alters microglial immunological phenotype one day after exposure

The main goal of this study was to characterize the effect of SPS on microglial immunological phenotype. The data demonstrated a significant decrease in microglial IL‐10 and TNF‐α gene expression 24 hr after SPS‐exposure (Figure 2). While expression for both of these genes decreased, SPS‐exposed rats exhibited a significantly higher TNF‐α:IL‐10 ratio compared to Controls (Figure 6a), indicating that SPS had a greater impact on IL‐10 gene expression than it did on TNF‐α expression. While there was no change in the absolute values of i‐NOS and ARG gene expression, a significant difference was found in the i‐NOS:ARG ratio, indicating that the balance between these two genes shifted significantly in the first 24 hr following SPS exposure toward a predominance of i‐NOS gene expression (Figure 6a). These results indicate somewhat of a mixed response of the microglia following SPS exposure. The decrease in IL‐10 gene expression and the increase of the TNF‐α:IL‐10 and i‐NOS:ARG ratios each indicate a shift toward a pro‐inflammatory phenotype. However, the decrease in TNF‐α gene expression is more consistent with an anti‐inflammatory phenotype. Finally, the lack of effect upon CD74 expression, MRC expression, and the ratio between these two genes shows no change toward either a pro‐ or anti‐inflammatory state. Overall, the data show that SPS does impact the microglia immunological phenotype, but the resulting change in activity (with respect to these six genes) cannot be clearly categorized as either pro‐inflammatory or anti‐inflammatory in nature.

These findings are in line with current opinions and findings that microglial responses to injury are complex and can vary greatly depending on the particular insult (Barakat & Redzic, 2016; Ransohoff, 2016). For several years, studies have applied the M1/M2 characterization scheme to microglial responses to injury. Both the M1 and the M2 immunological phenotypes have been suggested to have differing effects on injured tissues as well as increased expression of different characteristic genes (Cherry et al., 2014). For example, the M1 classification has been described as the “pro‐inflammatory” or “classical” phenotype that is associated with tissue damage and disease progression (Cherry et al., 2014). Genes often associated with the M1 phenotype include those that encode TNF‐α, CD74, and i‐NOS among many others (Hwang et al., 2017; Lisi et al., 2017; Zhang et al., 2018). Conversely, the M2 classification has been described as the “anti‐inflammatory” or “alternative” phenotype that is associated with tissue repair and injury resolution (Cherry et al., 2014). Genes often associated with the M2 phenotype include those that encode IL‐10, MRC, and ARG among others (Cherry et al., 2014; Lisi et al., 2017; Zhang et al., 2018). Unfortunately, while this classification system can be clearly demonstrated in vitro with macrophages, in vivo studies of microglia often reveal that responses in animal models cannot be so easily classified into two distinct groups (Ransohoff, 2016). The findings from our SPS model support this assertion, as we found differing results in each of the pairs of target genes that were analyzed.

### 4.3 The initial effect of SPS on microglial response is not identifiable three days after exposure

Typically, the behavioral changes induced by SPS are not expressed until 7 days after exposure (Souza et al., 2017), suggesting that the pathological mechanisms that underlie these changes develop earlier between Day 1 and Day 7. To assess the duration of the SPS‐induced effect upon microglial during this “development” phase, gene expression was also measured on Day 3 and Day 7. This evaluation did not reveal any differences between SPS‐exposed rats and Control rats in regard to the gene expression of the selected targets (Figures 3 and 4). These findings show that the SPS‐induced change in microglial immunological phenotype demonstrated on Day 1 is a short‐term change that resolves within 72 hr. However, it should be noted that this study only evaluated the gene expression of six select target genes. Evaluation of a broader range of genes may reveal changes that persist for longer than those reported in this study.

### 4.4 Previous exposure to SPS changes microglial response to CFC

To expand upon the characterization of the effects of SPS on microglial phenotype, we evaluated microglial gene expression after the SPS‐induced behavioral changes fully developed. A hallmark of the SPS model is impaired fear extinction memory expression that is observable 7 days after exposure (Knox et al., 2012, 2016; Souza et al., 2017). This behavioral change is typically demonstrated by subjecting SPS‐exposed rats to a CFC/Fear Extinction paradigm, and comparing how well they retain fear extinction memories compared to Controls (Knox et al., 2012, 2016; Souza et al., 2017). Therefore, gene expression was evaluated on Day 17 at the end of this “post‐development” phase following the completion of both extinction series. Our analyses revealed that IL‐10 expression was significantly decreased in the SPS‐exposed group compared to the Control group (Figure 5). The TNF‐α:IL‐10 ratio was also significantly elevated in the SPS group (Figure 6d), indicating a shift toward a pro‐inflammatory phenotype. While similar changes were also seen on Day 1, the duration of this change was notably different. More specifically, while the decrease in IL‐10 expression did not persist more than three days after SPS exposure, the decrease in IL‐10 appeared to be present for at least 10 days after CFC exposure in rats that were previously exposed to SPS. This indicates that previous exposure to SPS induced a change in the microglial response to CFC exposure. One possible explanation for this change is that the rats exposed to both SPS and CFC exhibited a prolonged decrease in microglial expression of IL‐10 and a persistent shift toward a pro‐inflammatory phenotype relative to rats that were exposed to CFC alone.

While it was out of the scope of this experiment to evaluate the functional impact of this decrease in IL‐10 gene expression, it is possible that this change in IL‐10 expression may play a role in the pathogenesis of PTSD. IL‐10 has been shown to decrease expression of pro‐inflammatory cytokines including IL‐2 and interferon‐γ (Taga & Tosato, 1992), IL‐1β (Herrera et al., 2019), and TNF‐α (Shin et al., 1999). IL‐10 also has been shown to decrease the pro‐inflammatory biomarker COX‐2 expression (Berg et al., 2001) and can interfere with the activity of the cytokine GM‐CSF (Hashimoto et al., 2001). While it should be noted that most studies that examine IL‐10 anti‐inflammatory activity have focused on peripheral immune cell populations, microglia have been shown to release IL‐10 and this release has been associated with a decrease in pro‐inflammatory cytokines (Cianciulli et al., 2015; Sun et al., 2019). Outside of this role as an anti‐inflammatory cytokine, IL‐10 has been shown to directly improve long‐term potentiation (LTP) (Nenov et al., 2019, 2019b), as well as reduce the impairment of LTP by pro‐inflammatory cytokines including IL‐1β (Kelly et al., 2001). As such, the decrease in IL‐10 in our study could be an indication that the rats exposed to SPS have a diminished capacity to form long‐term cortical memories.

This is relevant to PTSD pathogenesis as impaired fear extinction memory has been proposed as a basis of impairment of fear extinction memories in PTSD patients (Milad et al., 2008, 2009). PTSD patients have been shown to exhibit decreased activity of the prefrontal cortex, and a concurrent inability to maintain long‐term fear extinction memories that are formed in this part of the brain (Milad et al., 2008, 2009). The failure to maintain these memories is thought to prevent suppression of traumatic memories stored in the amygdala (Milad et al., 2009). The consequence of this dynamic is then expressed as a recurrence of fear memory over time. With diminished IL‐10 expression in frontal cortical microglia, it is possible that the decrease in IL‐10 will compromise long‐term extinction memory, and manifest as the recurring fear expression characteristic of PTSD.

### 4.5 IL‐10 expression levels are correlated with SPS‐induced behavioral changes

Our final objective of this study was to see whether the changes in microglial phenotype were related to changes in PTSD‐like behaviors. As discussed above, IL‐10 expression was most consistently impacted in this study, as it decreased in response to both the initial SPS exposure as well as showed a prolonged suppression following subsequent CFC. Moreover, as discussed above, decreased IL‐10 has the mechanistic potential for serving a role in the pathogenesis of PTSD. As such, we selected IL‐10 expression to do a *post hoc* comparison with a “fear persistence” index. Based on how this index was calculated (refer to Results above), low values indicated a persistence of fear expression (i.e., Day 15 fear expression was greater than Day 8 fear expression). Conversely, high values indicated resolution of fear expression (i.e., Day 8 fear expression was greater than Day 15 fear expression). Pearson Correlation revealed that there was a significant positive correlation between IL‐10 expression and the index, demonstrating that low IL‐10 values were associated with low index values. This indicates that as IL‐10 levels decrease, persistence of fear expression increases. This finding further supports the possibility of microglial IL‐10 expression being an important factor in PTSD pathogenesis, though future studies would need to be performed to show that there is a causal relationship between IL‐10 and persistent fear behavior.

## 5 CONCLUSIONS

Overall, this study demonstrated that SPS has a significant impact on the immunological phenotype of microglia of the frontal cortex. The response to SPS exposure could not be clearly defined as “pro‐inflammatory” or “anti‐inflammatory”, but a decrease in IL‐10 was consistently seen in both the development phase (Day 1 through Day 7) and the postdevelopment phase (Day 8 through Day 17) of the model. Furthermore, it was shown that while the initial response to SPS was short lived, exposure to a second stressful experience (CFC) could elicit a prolonged change in microglial immunological phenotype. This phenotype was characterized by a decrease in IL‐10 that was significantly correlated with persistence of fear expression. The findings of this study serve as the basis for future evaluation of the role of microglia populations in the pathogenesis of PTSD, which could become novel targets for future PTSD treatments and/or interventions.

## CONFLICT OF INTEREST

The authors of this work have no conflicts of interest to report.

## AUTHOR CONTRIBUTIONS

Cotrone, T.S. was the primary author and principle investigator of the study. Hocog, C.B., Ramsey, J.T., Sanchez, M.A., and Sullivan, H.M provided technical support. Scrimgeour, A.G. was the senior author and adviser, and co‐investigator of the study.

## DISCLAIMER

Research was approved by the Institutional Animal Care and Use Committee of the US Army Research Institute of Environmental Medicine and was conducted in compliance with the Animal Welfare Act and all other Federal Requirements. The views expressed are those of the authors and do not reflect the official policy of the Department of the Army, Department of Defense, or the U.S. Government.

### Peer Review

The peer review history for this article is available at https://publons.com/publon/10.1002/brb3.2011.

## Supporting information

Fig S1

Fig S2

Fig S3

Fig S4

Table S1

Table S2

## Data Availability

The data that support the findings of this study are available from the corresponding author upon reasonable request.
