# Supplementary figures and images for: Phenotypic characterization of frontal cortex microglia in a rat model of post‐traumatic stress disorder
Source: Brain Behav. 2021 Jan 12;11(3):e02011. doi: 10.1002/brb3.2011 (PMC7994680; doi:10.1002/brb3.2011)

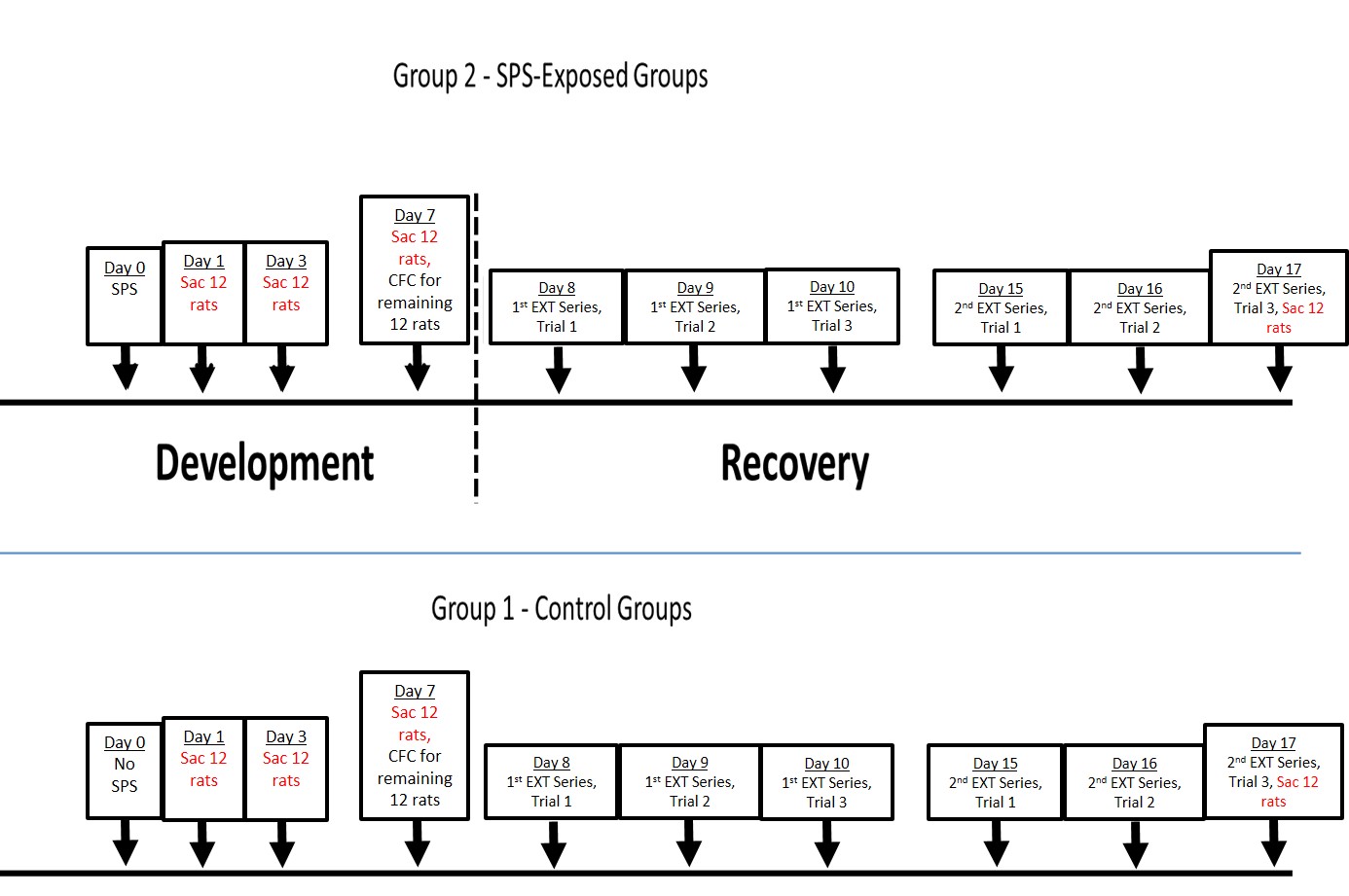

Supplement: Supplementary file 1 — Fig S1 [file BRB3-11-e02011-s001.jpg]

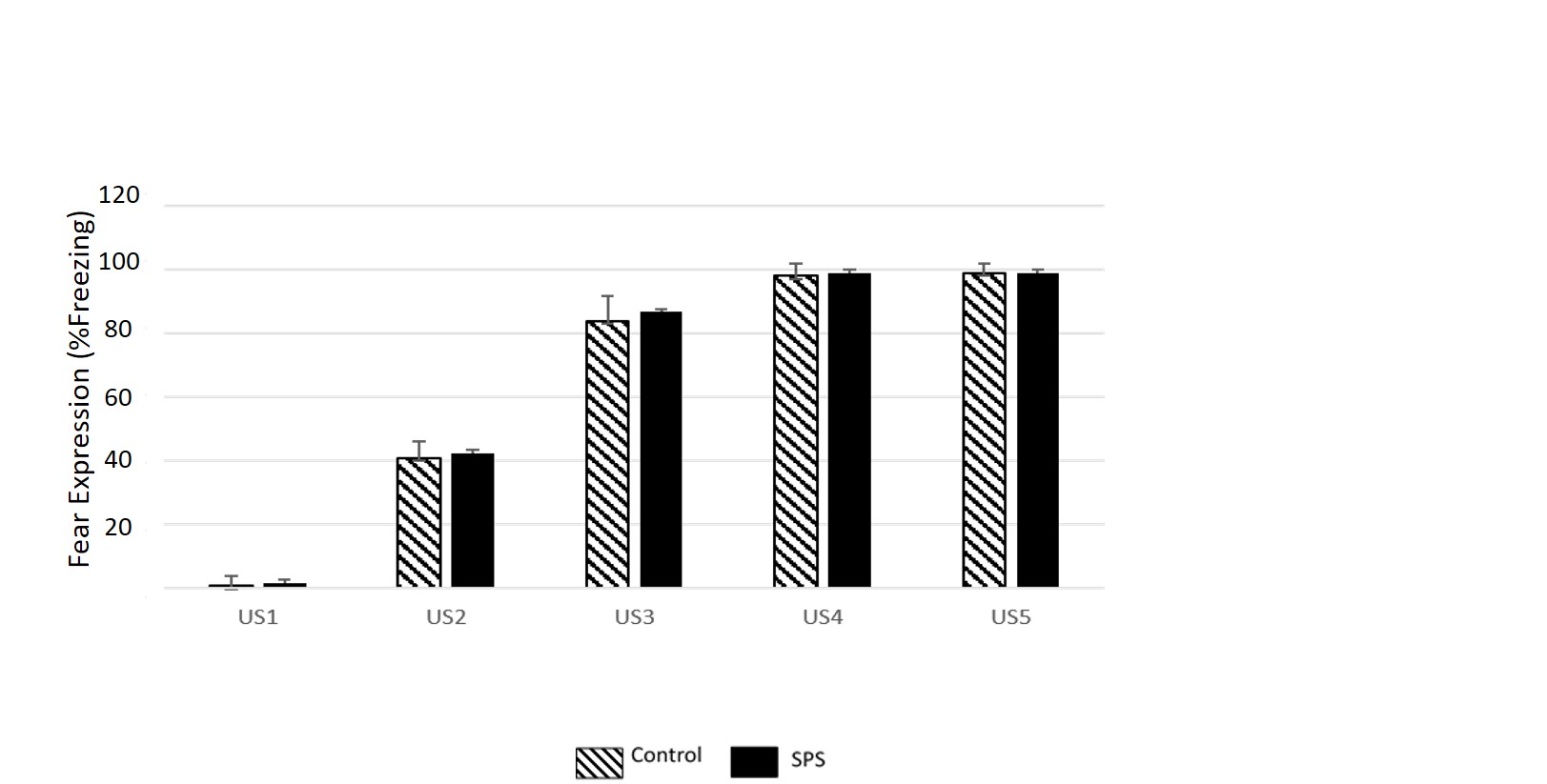

Supplement: Supplementary file 2 — Fig S2 [file BRB3-11-e02011-s003.jpg]

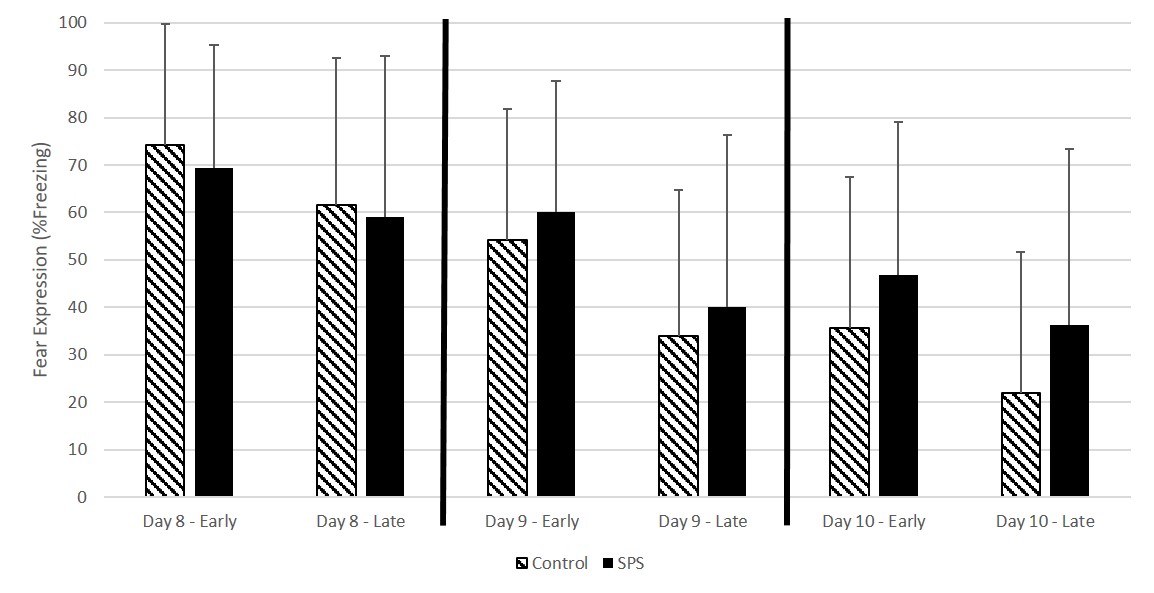

Supplement: Supplementary file 3 — Fig S3 [file BRB3-11-e02011-s002.jpg]

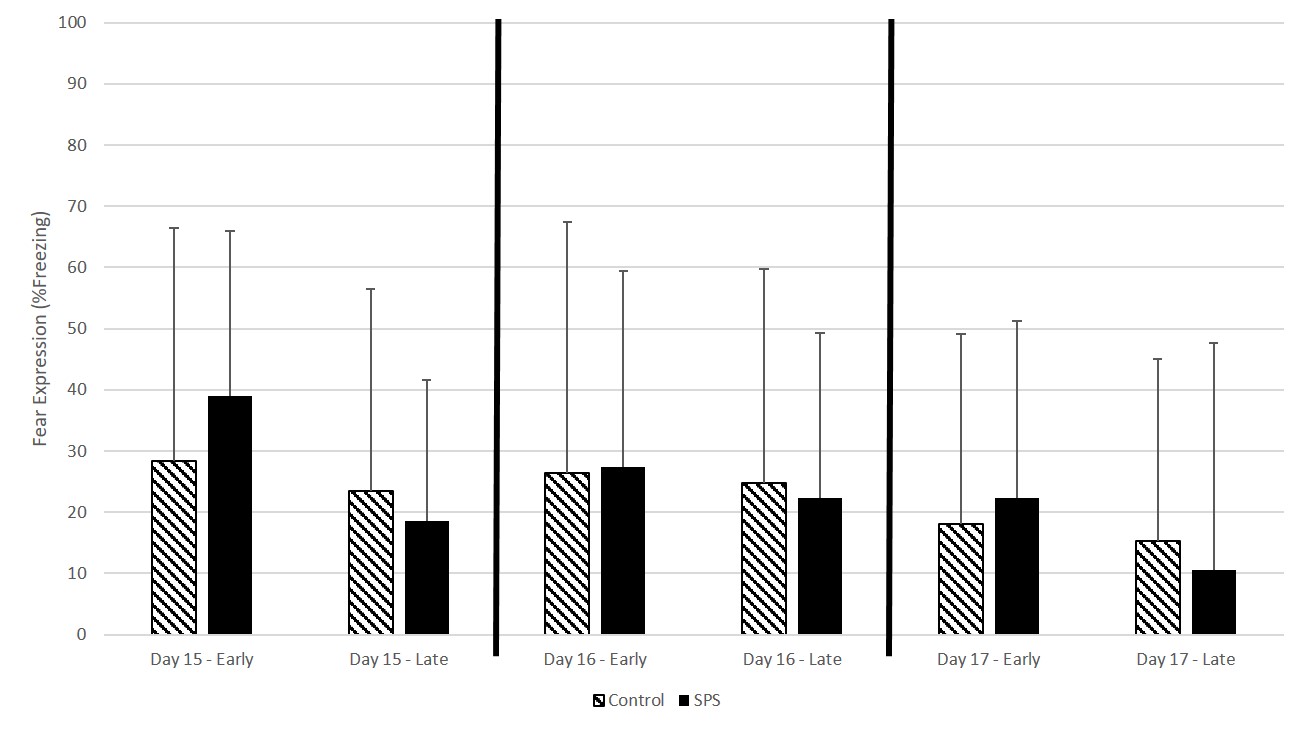

Supplement: Supplementary file 4 — Fig S4 [file BRB3-11-e02011-s005.jpg]

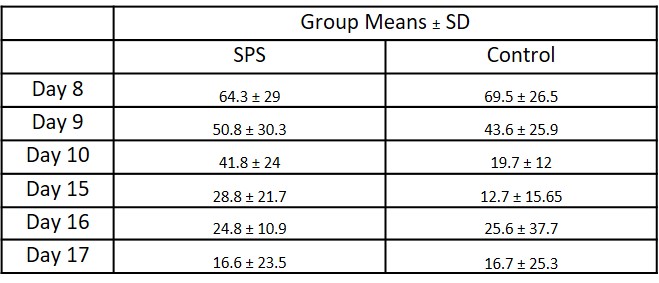

Supplement: Supplementary file 5 — Table S1 [file BRB3-11-e02011-s004.jpg]

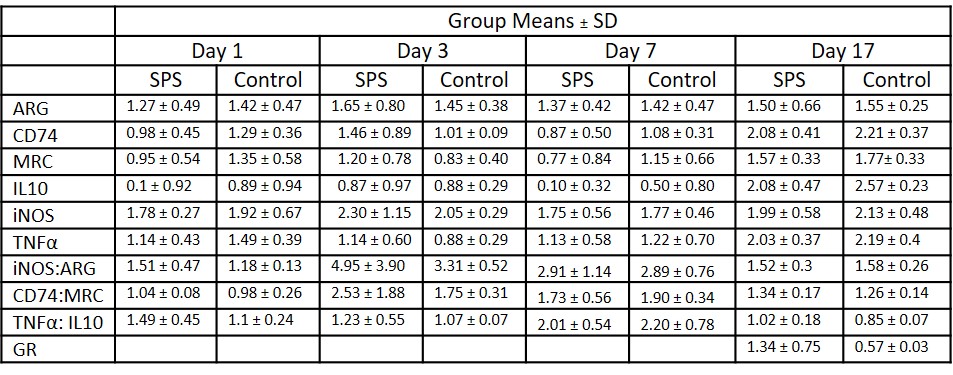

Supplement: Supplementary file 6 — Table S2 [file BRB3-11-e02011-s006.jpg]
